# Human immunodeficiency virus‐positive women's perspectives on breastfeeding with antiretrovirals: A qualitative evidence synthesis

**DOI:** 10.1111/mcn.13244

**Published:** 2021-07-13

**Authors:** Kan Man Carmen Li, Kan Yan Chloe Li, Debra Bick, Yan‐Shing Chang

**Affiliations:** ^1^ Guy's and St. Thomas' NHS Foundation Trust Evelina London Children's Hospital London UK; ^2^ Institute of Cardiovascular Science University College London London UK; ^3^ Warwick Clinical Trials Unit, Warwick Medical School University of Warwick Warwick UK; ^4^ Florence Nightingale Faculty of Nursing, Midwifery and Palliative Care King's College London London UK

**Keywords:** AIDS, antiretroviral prophylaxis, antiretroviral therapy, breastfeeding, HIV, qualitative evidence synthesis

## Abstract

Human immunodeficiency virus (HIV)‐positive women can breastfeed with minimal risk of mother‐to‐child transmission if taking antiretrovirals. Guidelines surrounding infant feeding for HIV‐positive women have evolved several times over the last two decades. Our review aimed to explore perspectives of breastfeeding with antiretrovirals from HIV‐positive women since the World Health Organization (2010) infant feeding and antiretroviral guidelines. HIV‐positive pregnant and postnatal women from all countries/settings were eligible. HIV‐positive women were either on an antiretroviral regimen at the time of the study, previously on an antiretroviral regimen, not initiated on a regimen yet, or enrolled in prevention of mother‐to‐child transmission (PMTCT) care. Quality assessment of all included studies were conducted. Four databases (CINAHL, EMBASE, MEDLINE and PsycINFO) were searched for studies conducted from January 2010 to October 2020. Nine papers were included in the review, of which two presented findings from the same study. Five analytical themes were developed via thematic synthesis: (1) awareness of breastfeeding with antiretrovirals, (2) turmoil of emotions, (3) coping mechanisms, (4) the intertwining of secret, stigma and support and (5) support needed. Support from family and health care professionals and coping approaches were important to overcome stigma and the emotional challenges of breastfeeding with antiretrovirals. Health care professionals should be familiar with the most updated national and local guidance surrounding infant feeding and antiretrovirals. Further research into interventions to encourage HIV‐positive women to adhere and commit to lifelong antiretroviral treatment (Option B+) for breastfeeding is required.

**Key messages**

- HIV‐positive women have a unique and challenging infant feeding experience, which requires tailored care and support.
- Health care professionals require updated knowledge of the most recent national and local infant feeding and antiretroviral guidelines for HIV‐positive women and need to communicate their advice effectively and consistently to HIV‐positive women.
- To increase social support for HIV‐positive women to breastfeed with antiretrovirals, community education and interventions are essential in low‐resource settings.
- Further research into HIV‐positive women's perspectives of breastfeeding with antiretrovirals in high‐resource countries is required.

## 1 INTRODUCTION

Breastfeeding offers many positive health benefits to the infant and breastfeeding woman, such as providing infants with immunity to disease, reduced risk of childhood obesity, and promoting mother‐infant bonding, and reduced risk of breast and ovarian cancers in women (World Health Organization [WHO], 2018). However, there are differences in infant feeding advice for HIV‐positive women between low‐ and high‐resource settings. Currently, the WHO and United Nations Children's Fund (UNICEF) (2016) promotes HIV‐positive women in low‐resource settings to exclusively breastfeed for 6 months whilst on lifelong antiretrovirals, introducing appropriate solids and non‐breast milk liquids from 6 months (complementary feeding) while continuing to breastfeed for up to 24 months and beyond, until a safe diet without breast milk can be provided. If the woman decides to stop breastfeeding completely, a 1‐month period of introducing solids and non‐breast milk liquids, with a gradual reduction of breast milk, is recommended. In high‐resource countries, where relevant resources (clean water, formula milk) are readily available, formula feeding is recommended instead to eliminate the risk of HIV mother‐to‐child transmission (MTCT) entirely (American Academy of Pediatrics, 2013; British HIV Association (BHIVA), 2020; World Health Organization, 2010). However, this is not to convey that breastfeeding with antiretroviral regimens have not occurred in high‐resource settings. For example, BHIVA (2020) recognises HIV‐positive women's free will in the United Kingdom to be able to decide to breastfeed, if specific criteria are met. Researchers have advocated for high‐resource settings to permit HIV‐positive women to breastfeed with antiretrovirals, in acknowledgement of people's capacity to understand the implications of doing so (Gamell, 2018; Kahlert et al., 2018).

Antiretrovirals enable HIV‐positive women to breastfeed with reduced risk of HIV MTCT (White et al., 2014) by lowering the risk of HIV MTCT during pregnancy and breastfeeding via suppressing the virus to below the detectable threshold (<50 copies per ml). Antiretroviral therapy (ART) involves lifelong use of three or more antiretrovirals (WHO & UNICEF, 2016). However, antiretroviral treatments do not fully eliminate the risk of HIV transmission via breast milk (Blumental et al., 2014), and it is apparent that antiretroviral regimens have different rates of HIV MTCT risk. For instance, Shapiro et al. (2014) found a 1.1% risk of HIV MTCT via exclusive breastfeeding (EBF) with maternal ART and a 1.7% risk for EBF with infant ART. Various antiretroviral regimens (Option A, B and B+ — see Table 1) exist for HIV‐positive women to breastfeed. HIV MTCT risk may also be elicited by breast infections, such as mastitis and cracked nipples (Zadrozny et al., 2017). Mixed feeding is the introduction of solids and other liquids with breast milk before the age of 6 months (WHO & UNICEF, 2016). Before 2010, mixed feeding was linked to an increased risk of HIV MTCT (Coovadia et al., 2007). Since 2010, research has indicated no difference in HIV MTCT risk between EBF and mixed feeding (Bispo et al., 2017). However, mixed feeding for HIV‐positive women is not an official recommendation and only a guiding practicing statement as it poses the risk of undermining EBF (WHO & UNICEF, 2016). With the absence of antiretrovirals, there is an HIV MTCT risk of 16% by breastfeeding (Nduati et al., 2000).

**TABLE 1:** Types of antiretroviral regimens

Regimen	Information	Guideline reference
Option A	• If an HIV‐positive woman's CD4 count is ≤350 cells/mm^3^, antiretroviral therapy (ART) is initiated for life, due to the HIV‐positive woman requiring treatment for her own health. • If an HIV‐positive woman's CD4 count >350 cells/mm^3^, a short‐term antiretroviral prophylaxis: ○ Azidothymidine (AZT), also known as zidovudine (ZDV), twice daily from 14 weeks' gestational age (GA) ○ Single dose of nevirapine (NVP) from the onset of labour with the initiation of daily AZT twice daily with lamivudine (3TC) for 1 week postnatally • Breastfeeding infant receives daily infant prophylaxis of NVP, initiated within 6–12 h from delivery, until 1 week after the end of the breastfeeding period. Infants who are not breastfeeding take NVP for 4–6 weeks. • WHO (2013) retracted its recommendation for Option A.	WHO (2010)
Option B	• If an HIV‐positive woman's CD4 count is ≤350 cells/mm^3^, antiretroviral therapy (ART) is initiated for life, due to the HIV‐positive woman requiring treatment for her own health. At the end of the breastfeeding period, the HIV‐positive woman can cease ART if CD4 count >350 cells/mm^3^, with continual monitoring for when CD4 count falls ≤350 cells/mm^3^ as an indicator to re‐start ART. • If an HIV‐positive woman's CD4 count is >350 cells/mm^3^, a triple antiretroviral prophylaxis is initiated from 14 weeks' GA, throughout delivery until 1 week after the end of the breastfeeding period. • Infant receives daily NVP or twice daily AZT from 6 to 12 h of delivery for 4–6 weeks, regardless of infant feeding method.	WHO (2010) WHO (2013)
Option B+	• The HIV‐positive woman initiates lifelong ART, consisting of triple antiretrovirals, initiated as early as HIV diagnosis and regardless of CD4 count. • Infant receives daily NVP or AZT for 4–6 weeks, regardless of chosen infant feeding method.	WHO (2013) WHO & UNICEF (2016)

In the last two decades, guidelines surrounding infant feeding practices for HIV‐positive women have been inconsistent due to conflicting research into HIV and prevention of mother‐to‐child transmission (PMTCT) care. HIV‐positive women breastfeeding with antiretrovirals has been a somewhat controversial area for policymakers, leading to multiple WHO guidelines and revisions of their recommendations over the last two decades (Dunkley et al., 2018) (see Appendix A). The uptake of guidelines has been varied across low‐resource settings due to dependence on local implementations and ongoing studies (WHO & UNICEF, 2016).

A range of barriers may deter HIV‐positive women on antiretrovirals to breastfeed and adhere to the antiretroviral regimen while breastfeeding, including HIV‐related stigma in community and health care settings (Flax et al., 2017), psychosocial challenges (Kaida et al., 2014), and inconsistent policies causing confusion (Nabwera et al., 2017). Research has found low continuity amongst postpartum HIV‐positive women to adhere to antiretroviral protocol, particularly postnatally (Nachega et al., 2012). Given this evidence, there are recommendations for further research into interventions to increase retention care, such as continuous engagement in PMTCT care, and adherence to antiretroviral regimens (Rollins et al., 2014; WHO & UNICEF, 2016).

Given the increasing accessibility of antiretrovirals for HIV‐positive women to breastfeed and extensive evolvement of infant feeding guidelines for HIV‐positive women, an understanding of the perspectives of breastfeeding with antiretrovirals from HIV‐positive women is important to inform health care practice by increasing awareness of specific care needs for this group of women and provide holistic support. The majority of clinical trials, studies and reviews focus on quantitative aspects, such as the efficacy of antiretrovirals, thus the need for this qualitative review.

## 2 METHODS

The review followed the guidelines provided by “Enhancing transparency in reporting the synthesis of qualitative research (ENTREQ).” The review was registered on the PROSPERO international prospective register of systematic reviews, with registration number: CRD42019136548. The review question was as follows: “What are HIV‐positive women's perspectives on breastfeeding with antiretrovirals?”

### 2.1 Eligibility criteria

Studies were included if participants were HIV‐positive pregnant and postnatal women. The HIV‐positive women may (a) have been undergoing an antiretroviral regimen at the time of the study; (b) have previously undergone an antiretroviral regimen and were not undergoing an antiretroviral regimen at the time of the study; or (c) have not initiated an antiretroviral regimen; or (d) have been enrolled under a PMTCT care/programme. The HIV‐positive women may or may not have previous experiences of breastfeeding. Any country/health care settings were eligible. Studies were included if they addressed factors of breastfeeding or infant feeding decision making with antiretrovirals amongst the above criteria. Studies only investigating HIV‐positive women's perspectives of breastfeeding irrespective of antiretrovirals were excluded. Studies that utilised WHO's antiretroviral and infant feeding guidelines for HIV‐positive women from 2010 onwards were eligible as this was the first instance of HIV‐positive women required to take lifelong antiretrovirals while breastfeeding or an antiretroviral prophylaxis lasting the entire breastfeeding period to one week post breastfeeding cessation. Antiretroviral regimens may include maternal and/or infant use of Option A, Option B or Option B+. Studies that were unclear about WHO guidelines and local guidelines for antiretroviral regimens and infant feeding recommendations at the time of the study were excluded.

The review included published qualitative primary research, not limited to designs such as grounded theory, phenomenology, ethnography and action research. Qualitative elements of mixed‐methods studies were included. Quantitative studies and quantitative elements of mixed‐methods studies were excluded. Grey literature, undergraduate and postgraduate dissertations were excluded as they were unpublished and may not have undergone a thorough peer‐review process. Reviews, policy documents, guidelines and opinion papers were excluded.

### 2.2 Search strategy

**FIGURE 1:**
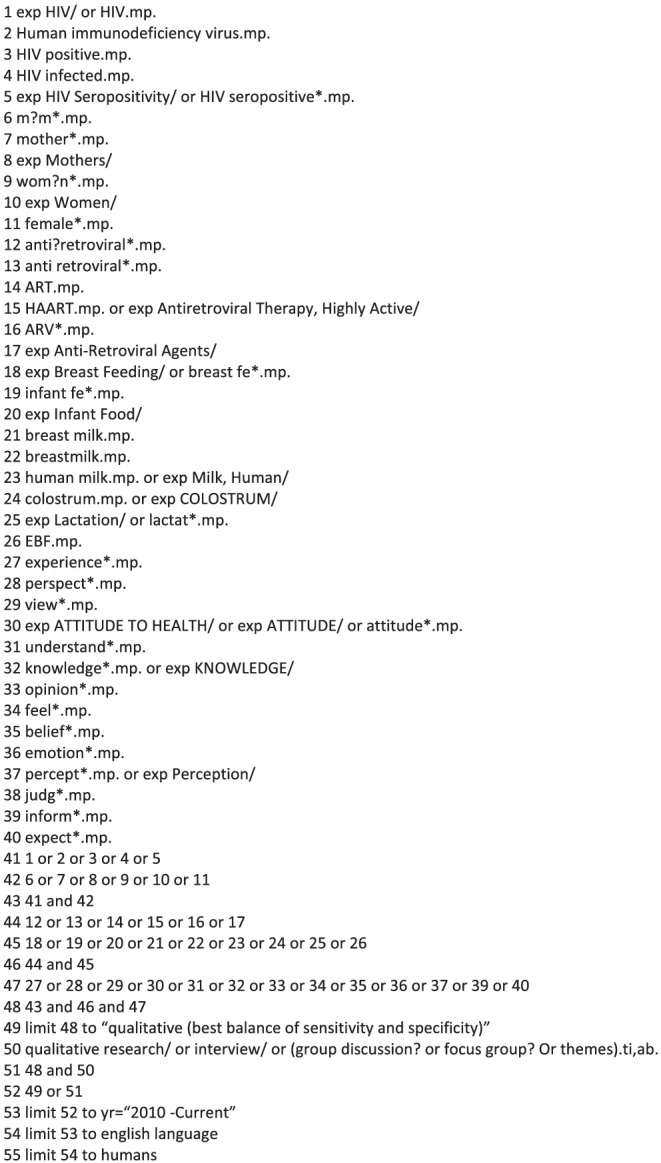
Example of electronic search strategy: Medline

A systematic search was performed by KMCL with Y‐SC's guidance on 31st January 2019 using four databases (CINAHL, MEDLINE, EMBASE and PsycINFO). MeSH terms and free terms with keywords and their variations included the following: (HIV AND mother*) AND (antiretroviral* AND breastfe*) AND [experience* OR perspective* OR view*]. Figure 1 shows an example of search history from one database. The electronic search was restricted from 2010. The search was updated on 10th October 2020. Only studies published in English language were included. Reference lists of previous relevant reviews (Laar & Govender, 2013; Nyoni et al., 2019; Tuthill et al., 2014) and included studies were hand searched. KMCL and Y‐SC performed a title‐abstract screen of the studies. The full text of the relevant studies was retrieved and screened for inclusion independently by KMCL and Y‐SC. Differences between screening results were resolved by discussion between KMCL and Y‐SC.

### 2.3 Quality assessment

Quality assessment for each included study was conducted independently by KMCL and Y‐SC using an adapted Critical Appraisal Skills Programme (CASP) (2018) 10‐item checklist for qualitative research (see Table 3). CASP question 10 was adapted from “How valuable was the research?” to “Is the research valuable?” to enable scoring. Each CASP question was answered via “Yes,” “No” or “Can't tell.” A “Yes” scored a value of 1. “No” and “Can't tell” scored 0. A maximum score of 10 could be achieved per paper. Differences between reviewers' assessments were resolved through discussion between KMCL and Y‐SC.

### 2.4 Data extraction and synthesis

Data were extracted on the study setting, aim, antiretroviral regimen HIV‐positive women received, infant feeding regimen in place at the time of the study, including whether free formula milk was provided to participants, the infant feeding method used by the participants, study design, data collection methods, data analysis and key findings. Data were synthesised using Thomas and Harden's (2008) three stages of thematic synthesis: (a) free line‐by‐line inductive coding, (b) development of descriptive themes from organising free codes, and (c) development of analytical themes. First, free line‐by‐line coding was carried out to form initial codes. Each finding was analysed and coded to reflect its content closely to the study's finding. Each finding could have more than one initial code applied. Afterwards, initial codes were compared with one another for similarities and differences, and grouped accordingly. Descriptive themes were formed to convey the meanings of groups of initial codes. Analytical themes were formed inductively to examine beyond the study's content. Data extraction and synthesis was conducted by KMCL, Y‐SC and KYCL. Final themes were agreed on by all authors.

### 2.5 Ethical considerations

This is a qualitative evidence synthesis and does not require an ethical approval. No funding was received to assist with the preparation of this article.

## 3 RESULTS

### 3.1 Characteristics of included papers

**FIGURE 2:**
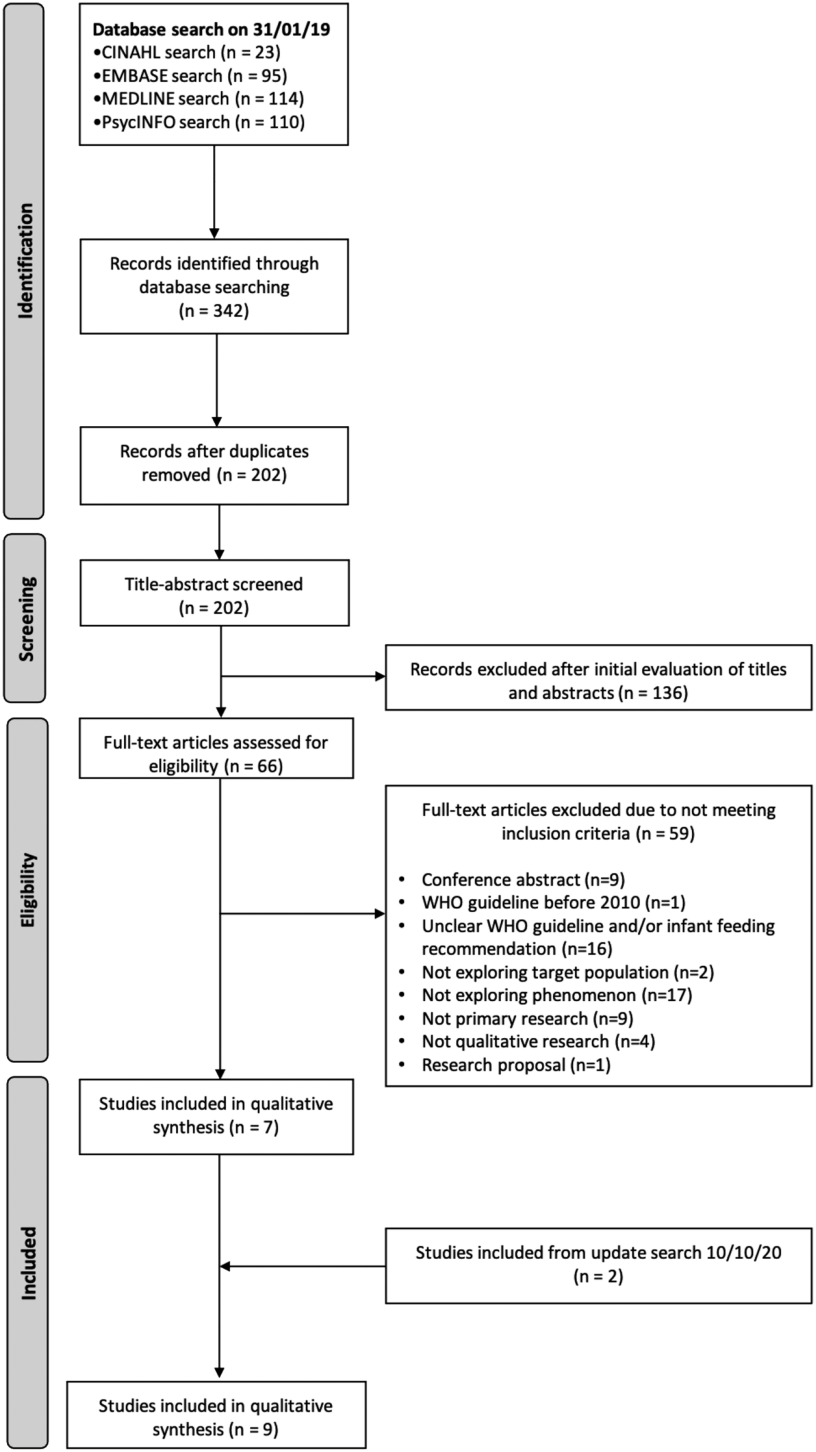
PRISMA summary of search

The initial search identified 202 citations after excluding duplicates. Sixty‐six papers from the initial search were screened by reading the full texts and evaluated for inclusion. Seven studies were identified from the original search. Two studies were included from the update search. Nine studies (Chadambuka et al., 2018; Croffut et al., 2018; Dunkley et al., 2018; Flax et al., 2017; Horwood et al., 2018; Katirayi et al., 2016; Matovu et al., 2014; Phakisi & Mathibe‐Neke, 2019; West et al., 2019) met the inclusion criteria, of which two were from the same study (Croffut et al., 2018; Flax et al., 2017). Figure 2 shows a summary of the screening process.

All the studies (*n* = 9) were conducted in African countries, including South Africa (Horwood et al., 2018; Phakisi & Mathibe‐Neke, 2019; West et al., 2019), Malawi (Croffut et al., 2018; Flax et al., 2017; Katirayi et al., 2016), Zimbabwe (Chadambuka et al., 2018) and Uganda (Dunkley et al., 2018; Matovu et al., 2014). HIV‐positive women experienced various stages of antiretroviral treatments and breastfeeding methods in relation to national and local infant feeding protocols and recommendations at the time of the study (see Table 2). All studies involved qualitative methodologies.

**TABLE 2:** Summary of included papers

Paper	Country	Aim	Antiretroviral (ARV) regimen	Infant feeding method/recommendation	Participants	Study design; sampling; data collection; analysis	Key results
Chadambuka et al. (2018)	Zimbabwe	To understand HIV‐positive women's perspectives and experiences of Option B+, in terms of its acceptability, usage of services, barriers and facilitators.	• Option A or Option B+ for more than 6 months. • WHO (2013) Option B+ guideline implemented by Zimbabwe's Ministry of Health and Child Care in 2014	• Breastfeeding recommended with Option B+	• Interviews: • 43 HIV‐positive pregnant or breastfeeding women • 5 key informants • FGDs: • 22 HIV‐positive women	• In‐depth interviews • FGDs • Thematic analysis	HIV‐positive women generally accepted Option B+. Although women were fearful of the lifelong commitment after breastfeeding cessation and side effects. They were concerned with having an insufficient food supply and being unable to ask questions during counselling sessions. Facilitators to Option B+: “receiving a simplified pill regimen,” “ability to continue breastfeeding beyond 6 months like HIV‐negative women,” “partner, community and health worker support.” Barriers to Option B+: “distance of health facility,” “non‐disclosure of HIV status,” “poor male partner support” and “knowing someone who had a negative experience on antiretroviral therapy (ART).”
Croffut et al. (2018)^a^	Lilongwe District, Malawi	To explore HIV‐positive women's perceptions of how body size affects infant feeding decisions and identify differences in these perceptions between HIV‐positive women under ART and those lost to follow up.	• 32 women enrolled under Option B+. • 32 women lost to follow up.	• Malawi followed WHO (2010) recommendation for exclusive breastfeeding (EBF) for 6 months but modified recommendation by extending the continuation of breastfeeding from 12 to 24 months.	• 64 HIV‐positive women	• In‐depth interviews • Thematic content analysis	Over 80% HIV‐positive women favoured larger body types to be healthier and overweight women more able to breastfeed. Approximately 50% presumed an underweight woman could breastfeed. Body size perceptions influenced HIV‐positive women's perception of their ability to breastfeed. Some HIV‐positive women emphasised body size was not an issue to breastfeed and to follow advice from health care professionals.
Dunkley et al. (2018)	Uganda	To understand the infant feeding experiences of women living with HIV, in amidst of continuously changing infant feeding guidelines for HIV‐positive women.	• All women received ART (Option B+). • Uganda followed WHO (2010) HIV and Infant Feeding guideline and WHO (2013) guideline.	• Ugandan national recommendation for women living with HIV to practice EBF for 6 months and continue breastfeeding until 12 months with complementary feeding.	• 20 postpartum Ugandan HIV‐positive women	• Semistructured interviews • Content analysis	Woman's infant feeding decisions were not in line with the WHO infant feeding guidelines due to three reasons: “perception of conflicting recommendations regarding infant feeding,” “fear of prolonged infant HIV exposure through breastfeeding” and “social and structural constraints shaping infant feeding decision‐making.”
Flax et al. (2017)^a^	Lilongwe District, Malawi	To identify what facilitators and barriers influenced HIV‐positive women to adhere to Option B+, and how this affected breastfeeding duration.	• 32 women undergoing ART (Option B+). • 32 lost to follow up from ART (Option B+).	• Various statuses of breastfeeding. • Malawi followed WHO (2010) recommendation to EBF for 6 months but modified recommendation by extending the continuation of breastfeeding from 12 to 24 months.	• 64 HIV‐positive women	• Qualitative in‐depth interviews • Purposive sampling • Thematic analysis	Better experiences of social support were found in women on Option B+ than those loss to follow up (LTFU). Reasons for LTFU: “fear of HIV disclosure,” “anticipated or experienced stigma,” “insufficient social support,” “non‐acceptance of HIV status,” “ART side effects,” “lack of funds for transport,” “negative experiences with clinic staff.” LTFU women continued to breastfeed due to the benefits of breast milk, despite no longer on Option B+ and fear of HIV MTCT.
Horwood et al. (2018)	KwaZulu‐Natal, South Africa	To discover the experience of infant feeding decision making in HIV‐positive women in rural and urban areas of South Africa.	• Undergoing ART.	• 9 intended to EBF. • 2 intended to exclusively formula feed (EFF). • Variable breastfeeding statuses throughout longitudinal study.	• 11 HIV‐positive women	• Qualitative longitudinal cohort design • Purposive sampling • In‐depth interviews over 6 months post‐delivery	3 major themes: “the role of health care workers in provision of infant feeding advice,” “fear of HIV transmission influences feeding choices” and “importance of mothers' self‐efficacy to resist pressures from family and community to change her feeding practice.”
Katirayi et al. (2016)	Malawi	To identify the facilitators and barriers which influenced pregnant and postpartum HIV‐positive women to begin and adhere to Option B+.	• Women were under Option B+, consisting of tenofovir, lamivudine and efavirenz.	• After Malawi implemented Option B+ in July 2011, breastfeeding recommendations involved EBF for 6 months followed by complementary feeding for up to 2 years postpartum. • All 20 HIV‐positive postpartum women had breastfed.	• In‐depth interviews: • 19 HIV‐positive pregnant women • 20 HIV‐positive postpartum women • For FGDs: • 4 health care workers • 4 HIV‐positive pregnant women	• In‐depth interviews • FGDs • Thematic analysis	Women's acceptability of the drug regimen: “perception of drug as health‐enhancing,” “normalization of appearance and infant feeding practice” and “scepticism about lifelong treatment.” Women's acceptability of the PMTCT counselling services: “inappropriate timing of ART initiation,” “poor counselling procedures,” “loss to follow up.”
Matovu et al. (2014)	Kampala, Uganda	To understand HIV‐positive women's and other key figures' views on the WHO, 2010 infant feeding guidelines.	Uganda Ministry of Health uptake of the WHO (2010) recommendation of one of the following: (1) Maternal zidovudine (ZDV) during pregnancy with daily NVP for infant after delivery and until the end of breastfeeding. (2) Triple ARV regimen during pregnancy and continue until end of breastfeeding. (3) Initiation of lifelong triple ARV drugs regardless of CD4 count during pregnancy after known HIV status.	• Recommendation of EBF for first 6 months of life.	• 9 HIV‐positive pregnant women • 10 HIV‐positive postpartum women • 10 HIV‐positive peers • 10 male partners • 10 family members of pregnant women • 12 key informants	• FGDs • Interviews • Purposive sampling	Most participants, except for male partners, were favourable of EBF. Health care professionals were largely in favour of complementary feeding at 6 months while continuing to breastfeed, however other participants were not. Most of the participants preferred HIV‐positive women to take ARVs while breastfeeding instead of the infant taking ARVs.
Phakisi and Mathibe‐Neke (2019)	Mangaung, South Africa	To investigate HIV‐positive women's exclusive breastfeeding experience in the first 6 months after birth.	• Enrolled in PMTCT care and attended antenatal clinic. • Specific ARV treatment of the participants was not stated at the time of the study but under WHO (2010) guideline, implemented via the Tshwane Declaration in August 2011	• South Africa followed WHO (2010) advice to adopt a single feeding practice for HIV‐positive women. Exclusive breastfeeding promoted. • 15 HIV‐positive women chose to EBF for 6 months • Free formula milk was discontinued in South Africa in 2011.	• 15 HIV‐positive women	• Purposive sampling • Individual unstructured in‐depth interviews • Thematic data analysis	HIV‐positive women had positive and negative experiences with exclusive breastfeeding. Experiences with EBF were shaped by socio‐cultural factors and health care professionals.
West et al. (2019)	Johannesburg, South Africa	To explore HIV‐positive and negative women's breastfeeding decision making.	• Enrolled in “FRESH Start” program at Witkoppen Health and Welfare Centre involving HIV testing from first antenatal appointment and immediate ART for HIV‐positive women. • HIV‐positive women's infant tested for HIV at 6 weeks. • 22 HIV‐positive women on ART.	• Witkoppen Health and Welfare Centre implemented the Department of Health Infant and Young Child Feeding Policy (2013) of EBF for 6 months, breastfeeding and complementary feeding until 12 months for women on ART, and women to continue to breastfeed up till 24 months. • 12 HIV‐positive women breastfeeding • 10 HIV‐positive women formula feeding.	• 22 HIV‐positive women on ART • 12 health care providers	• Semi‐structured in‐ depth interviews • Content analysis	HIV‐positive pregnant women were less keen on the idea of breastfeeding and less likely to EBF in the first 6 months compared to HIV‐negative women. Factors that affected women to breastfeed included conflicting advice from infant feeding counsellors, social and economic influences, and fear of HIV transmission.

^a^ Croffut et al. (2018) and Flax et al. (2017) report the same study.

Quality assessment of included papers (see Table 3) results in only one study scoring a total of 10 (Dunkley et al., 2018). Matovu et al. (2014) scored the lowest with 6. Many studies failed to address the relationship between the researchers and participants, which reduced the credibility of these studies. Some studies did not provide sufficient evidence in their methodology for a rigorous data analysis and recruitment strategy.

**TABLE 3:** Quality assessment of included studies

Paper	1. Clear aim?	2. Appropriate qualitative methodology?	3. Appropriate research design to address aims?	4. Appropriate recruitment strategy to aims of research?	5. Data collection addressed research issues?	6. Consideration of relationship between researcher and participants?	7. Consideration of ethical issues?	8. Rigorous data analysis?	9. Clear statement of findings?	10. Was the research valuable?	CASP score (/10)
Chadambuka et al. (2018)	Yes	Yes	Yes	Yes	Yes	Can't tell	Yes	Yes	Yes	Yes	9
Croffut et al. (2018)	Yes	Yes	Yes	Yes	Yes	Can't tell	Yes	Yes	Yes	Yes	9
Dunkley et al. (2018)	Yes	Yes	Yes	Yes	Yes	Yes	Yes	Yes	Yes	Yes	10
Flax et al. (2017)	Yes	Yes	Yes	Yes	Yes	Can't tell	Yes	Yes	Yes	Yes	9
Horwood et al. (2018)	Yes	Yes	Yes	Yes	Yes	Can't tell	Yes	Yes	Yes	Yes	9
Katirayi et al*. (* 2016)	Yes	Yes	Yes	Yes	Yes	Can't tell	Yes	Yes	Yes	Yes	9
Matovu et al. (2014)	Yes	Can't tell	Can't tell	Can't tell	Yes	No	Yes	Yes	Yes	Yes	6
Phakisi and Mathibe‐Neke (2019)	Yes	Yes	Yes	Can't tell	Yes	No	Yes	No	Yes	Yes	7
West et al. (2019)	Yes	Yes	Yes	Can't tell	Yes	No	Yes	Yes	Yes	Yes	8

### 3.2 Themes

Five analytical themes were generated from the included papers: (1) awareness of breastfeeding with antiretrovirals, (2) turmoil of emotions, (3) coping mechanisms, (4) the intertwining of secret, stigma and support and (5) support needed.

#### 3.2.1 Awareness of breastfeeding with antiretrovirals

HIV‐positive women had variable levels of knowledge on the infant feeding guidelines recommended for HIV‐positive women at the time of the study, risks of breastfeeding with antiretrovirals and general benefits of breastfeeding.

##### Infant feeding protocol

Some HIV‐positive women were aware of the infant feeding protocol concurrent with infant feeding guidelines for HIV positive women at the time (Flax et al., 2017; Horwood et al., 2018; Matovu et al., 2014; Phakisi & Mathibe‐Neke, 2019). For instance, HIV‐positive women knew they were recommended to exclusively breastfeed for 6 months with no introduction of solids and non‐breast milk liquids (Horwood et al., 2018; Matovu et al., 2014). Few HIV‐positive women knew the importance of adherence to antiretrovirals while breastfeeding to reduce the risk of HIV MTCT (Flax et al., 2017; Horwood et al., 2018; West et al., 2019). On the other hand, a minority were unaware of updated infant feeding guidelines for HIV‐positive women, such as not knowing HIV‐positive women could breastfeed with antiretrovirals as it was not previously promoted (Dunkley et al., 2018; Horwood et al., 2018). For example, one woman said: “No, I didn't know I was going to breastfeed as I thought only [HIV] negative people were allowed to breastfeed, until I visited the clinic” (Horwood et al., 2018, p. 4).

##### Risks of breastfeeding with antiretrovirals

Many HIV‐positive women understood the risk of HIV MTCT from breastfeeding with Option B+ but were unable to explain how HIV transmission occurs (Dunkley et al., 2018). Few HIV‐positive women were aware of factors that could increase the risk of HIV MTCT while breastfeeding with antiretrovirals, such as breast sores (Horwood et al., 2018) and infant thrush (Horwood et al., 2018). Although mixed feeding has been found to have no difference in HIV MTCT compared with EBF, some women were only promoted EBF for 6 months. These women thought mixed feeding before 6 months could increase HIV MTCT (Horwood et al., 2018; Phakisi & Mathibe‐Neke, 2019). A South African woman reported: “… I was told several times at the clinic that it should either be breast milk or formula alone, that breast milk was best and never to mix feed because babies can contract HIV and other infection …” (Phakisi & Mathibe‐Neke, 2019, p. 30).

##### Benefits of breastfeeding

HIV‐positive women's awareness of breastfeeding benefits influenced their decisions to continue breastfeeding as they desired their infant to acquire the benefits of breastfeeding (Flax et al., 2017; Horwood et al., 2018; Phakisi & Mathibe‐Neke, 2019). A South African woman reported, “He gets healthy; he is protected from diseases. Breast milk is healthy” (Horwood et al., 2018, p. 6). Another HIV‐positive woman from Malawi continued breastfeeding after stopping antiretrovirals despite the recommended infant feeding policy (Flax et al., 2017).

#### 3.2.2 Turmoil of emotions

The decision making and experiences of breastfeeding antiretrovirals were challenging for HIV‐positive women, leading to an overwhelming weight of emotions conveyed by the women.

##### Striving for motherhood with influences of personal experiences and intuition

HIV‐positive women wanted to breastfeed in order to fulfil their innate desire to achieve motherhood (Horwood et al., 2018). Furthermore, the decision to breastfeed or not was reinforced by previous breastfeeding experiences of other children, such as previously breastfeeding their older children encouraged them to breastfeeding their baby (Horwood et al., 2018) or having had an emotionally challenging experience with caring for a child with HIV deterred HIV‐positive women from breastfeeding (Dunkley et al., 2018). At times, a mother's instinct influenced them to stop breastfeeding and starting formula or replacement feeding due to experiences of low breast milk supply not meeting infant's feeding demand and poor infant weight gain (Dunkley et al., 2018; Horwood et al., 2018; Matovu et al., 2014; Phakisi & Mathibe‐Neke, 2019). One woman said:

I started giving my baby formula at one month because he would not stop crying. So I believed he was not getting enough milk. ( Phakisi & Mathibe‐Neke, 2019, p. 31)

##### Pressured to breastfeed

A strong sense of frustration was reported by HIV‐positive women over pressure from health care professionals to breastfeed (Dunkley et al., 2018; Horwood et al., 2018). Additionally, the pressure to initiate and continue breastfeeding stemmed from their partners, family members and communities (Dunkley et al., 2018; Horwood et al., 2018; West et al., 2019). For example, one woman reported “...when I produced him [gave birth], immediately I never breastfeed him. The man begged me, ‘please breastfeed the baby’.” (Dunkley et al., 2018, p. 7). Mixed feeding was instigated upon women from family and communities due to perceptions of breast milk alone being inadequate to meet the infant's feeding demands (Horwood et al., 2018; Phakisi & Mathibe‐Neke, 2019).

##### Fear of HIV MTCT

Some HIV‐positive women were reluctant to breastfeed due to fear of HIV MTCT from breastfeeding with antiretrovirals and mixed feeding before 6 months. Many HIV‐positive women decided to formula feed to avoid breastfeeding entirely (Dunkley et al., 2018; Horwood et al., 2018; West et al., 2019).

I didn't want any chance for them [the infant] to get HIV. I felt that they [health care providers] said if you are positive and take your medication properly, then you can have a negative baby. I decided that I don't want any chance. (West et al., 2019, p. 4)

On the other hand, HIV‐positive women who were breastfeeding with antiretrovirals (Dunkley et al., 2018; Horwood et al., 2018; Phakisi & Mathibe‐Neke, 2019; West et al., 2019) or starting to introduce solids and non‐breast milk liquids while breastfeeding (Dunkley et al., 2018) felt fearful of the risk of HIV MTCT. This fear invoked HIV‐positive women wanting to stop breastfeeding and offer replacement feeding before 6 months (Flax et al., 2017; Horwood et al., 2018).

For HIV‐positive women who were advised to EBF for 6 months only, they were conscious of avoiding mixed feeding as they believed it poses a greater risk of HIV MTCT (Horwood et al., 2018; Phakisi & Mathibe‐Neke, 2019). Fear of HIV MTCT was deduced to feelings of guilt and self‐blame if their infant contracted HIV (West et al., 2019). Alleviation of this fear would only occur after receiving negative infant HIV test results (Dunkley et al., 2018; Phakisi & Mathibe‐Neke, 2019). In one instance, a South African HIV‐positive woman made the conscious choice to mixed feed (reasons for this choice were not described), despite being aware of the risk of MTCT and felt guilty of her choice (Phakisi & Mathibe‐Neke, 2019). She stated: “I am even afraid of taking him for the test as I know I mixed fed him. I already feel guilty about what I did, and I pray every day that my child should be HIV‐negative” (Phakisi & Mathibe‐Neke, 2019, p. 30).

##### Financial and lifestyle stress

Many HIV‐positive women struggled to afford formula milk and had no choice but to breastfeed (Dunkley et al., 2018; Horwood et al., 2018; West et al., 2019). As a woman reported, “I chose to breastfeed because I am not working and sometimes you may find that I would not have money to buy formula as the father also doesn't have a good job” (West et al., 2019, p. 5). This stress persisted during the period of introducing solids and other non‐breast milk liquids while gradually stopping breastfeeding when trying to afford replacement feeding (Flax et al., 2017; Horwood et al., 2018). A Ugandan woman's partner begged her to breastfeed due to financial constraints (Dunkley et al., 2018). In contrast, some HIV‐positive women decided against breastfeeding or stopped breastfeeding to work/return to work or seek employment for financial stability (Dunkley et al., 2018; Horwood et al., 2018; Phakisi & Mathibe‐Neke, 2019; West et al., 2019). Dunkley et al. (2018) reported that HIV‐positive women felt frustrated with the need for regular infant HIV testing if they continued to breastfeed with Option B+.

##### Side effect struggles

Experiences of antiretroviral side effects from HIV‐positive women, such as dizziness, nausea, vomiting and upset stomachs, deterred HIV‐positive women from breastfeeding with antiretrovirals (Flax et al., 2017; Matovu et al., 2014) and unreadiness to commit to lifelong therapy (Option B+) (Flax et al., 2017; Katirayi et al., 2016). However, HIV‐positive women undergoing Option B+ did not experience the severity of side effects as much (Chadambuka et al., 2018). For example, one lactating woman from Zimbabwe reported:

I wanted to say that we accept it well because lifelong antiretroviral therapy keeps us healthy all the time and we are able to do our daily chores without being sick all the time since we will be taking our medication well. (Chadambuka et al., 2018, p. 5)

#### 3.2.3 Coping mechanisms

A turmoil of emotions mentioned above over their infant's risk of HIV transmission and infant feeding experience led to coping behaviours exhibited by HIV‐positive women to withstand their challenging infant feeding experiences.

##### Emotional resilience

HIV‐positive women commented on the need for emotional resilience while breastfeeding with antiretrovirals and making infant feeding decisions independently (Horwood et al., 2018). For example, “No, I haven't discussed anything because I am a strong person, I've told myself that I am going to breastfeed so there is no one that I fought with or say something” (Horwood et al., 2018, p. 9). Emotional resilience was often reported by HIV‐positive women in other instances, such as when stopping breastfeeding to deal with the emotionally challenging experience from being unable to provide breast milk (Horwood et al., 2018), adhering to antiretroviral medications while breastfeeding (Chadambuka et al., 2018) and disregarding family members pressuring them to mixed feed (Horwood et al., 2018).

##### Turning to religious beliefs

A minority of HIV‐positive women resorted to religion as a method to cope with their emotional struggles and fear over the risk HIV MCT while breastfeeding: “I used to pray to God to be the one to protect him … So that he does not get infected with HIV … because of breastfeeding....” (Dunkley et al., 2018, p. 7). Similarly, a South African woman prayed in anticipation for her infant's HIV test as she had mixed fed her baby (Phakisi & Mathibe‐Neke, 2019).

#### 3.2.4 The intertwining of secret, stigma and support

Many HIV‐positive women concealed their infant feeding method to conceal their HIV‐positive status due to a strong social stigma prevalent in communities towards infant feeding and HIV. Having a strong support network, including family and health care professionals, provided HIV‐positive women a better infant feeding experience.

##### Interlinks between (non)disclosure of HIV status and social support

The support received by HIV‐positive women varied and was dependent on whether they disclosed their HIV status to their family. Socio‐cultural expectations from peers and family members to follow community infant feeding practices to avoid disclosing their HIV status were reported by HIV‐positive women who were pressured to breastfeed (Dunkley et al., 2018; West et al., 2019) or continued breastfeeding longer than they wanted to (Dunkley et al., 2018; Horwood et al., 2018) or initiate mixed feeding (Horwood et al., 2018; Phakisi & Mathibe‐Neke, 2019; West et al., 2019). Some HIV‐positive women stopped breastfeeding due to concerns that their family members would initiate mixed feeding while they were not present as they were informed to avoid mixed feeding as per local recommendations at the time of the study (Horwood et al., 2018; Phakisi & Mathibe‐Neke, 2019). Nondisclosure of their HIV status led one South African woman to deceive her family by giving “insufficient breast milk” as a reason to commence formula feeding (West et al., 2019). HIV‐positive women on Option B+ were reported to experience less stigma because they could breastfeed beyond 6 months and assimilate to cultural breastfeeding norms like noninfected individuals (Chadambuka et al., 2018; Flax et al., 2017; Katirayi et al., 2016), as this quote illustrated: “… nowadays no one can tell the difference between an HIV‐positive and an HIV‐ negative person. We all look the same...” (Chadambuka et al., 2018, p. 5).

In contrast, some HIV‐positive women who disclosed their HIV status received support from their partners and family to breastfeed and follow their local infant feeding recommendations at the time, such as avoiding mixed feeding (Dunkley et al., 2018; Horwood et al., 2018). One South African HIV‐positive woman openly shared her HIV‐status to family and strangers and explained how she was exclusively breastfeeding with antiretrovirals (Phakisi & Mathibe‐Neke, 2019). “I disclosed my status to everyone. I talk a lot about it, even to strangers. Some people used to comment about my breastfeeding while I was HIV‐positive. I would tell them that my child was safe because I was taking antiretrovirals and not mixed feeding” (Phakisi & Mathibe‐Neke, 2019, p. 30). Another South African woman believed her choice to exclusively breastfeed has nothing to do with her family (Horwood et al., 2018).

##### The value of health care professionals

Some HIV‐positive women relied on health care professionals to recommend how they should feed their infants or wanted to make decisions with health care professionals (Croffut et al., 2018; Horwood et al., 2018). For instance, HIV‐positive women actively sought infant feeding advice from health care professionals when they were unsure of when to stop breastfeeding: “I'm waiting for Monday to go for 6 months [clinic] visit, then they will tell me what to do” (Horwood et al., 2018, p. 4). HIV‐positive women gained awareness of current infant feeding protocol for HIV‐positive women and the importance of adherence to antiretroviral regimen from health care professionals at the time of the study (Flax et al., 2017; Horwood et al., 2018; West et al., 2019). Some HIV‐positive women only became aware they could breastfeed via antiretrovirals after consulting with health care professionals (Horwood et al., 2018). HIV‐positive women also received general infant feeding advice, such as health and bonding benefits of breastfeeding (Horwood et al., 2018), expressing and storing breast milk (West et al., 2019) and avoidance of mixed feeding as per local recommendations (Horwood et al., 2018; Phakisi & Mathibe‐Neke, 2019). Some HIV‐positive women were motivated by health care professionals to adhere to antiretrovirals to reduce the risk of HIV MTCT while breastfeeding (Horwood et al., 2018).

#### 3.2.5 Support needed

To neutralise HIV‐positive women's challenging experience of breastfeeding with antiretrovirals, two areas of support needs were described by the women.

##### Role models

HIV‐positive women wanted role models of other HIV‐positive women breastfeeding with antiretrovirals to share their experiences and support, to encourage uptake of antiretrovirals, particularly for HIV‐positive women who opted for formula feeding (Chadambuka et al., 2018; West et al., 2019).

Yes, it encourages, because if you hear from their experiences, you learn from what they would have gone through, how it helped them, where they came from and where they are going. (Chadambuka et al., 2018, p. 4)

##### Enhancing education provision to women and their support network

The need for further education and consolidation of education offered by health care professionals was highlighted by HIV‐positive women. Confusion amongst HIV‐positive women regarding infant feeding advice they received was frequently reported, such as the conflict between breast is best and the HIV MTCT risk associated with breastfeeding with Option B+ (Dunkley et al., 2018). Updated infant feeding recommendations in line with current national and local guidelines are required by HIV‐positive women as there were inconsistencies surrounding advice on breastfeeding cessation and introducing complementary foods from 6 months (Dunkley et al., 2018; Horwood et al., 2018; Phakisi & Mathibe‐Neke, 2019; West et al., 2019), and a few HIV‐positive women received outdated advice (Horwood et al., 2018; Phakisi & Mathibe‐Neke, 2019). For example:

Breastfeeding was a very good experience for me. I became very sad when I had to discontinue the practice. I wanted to continue, but was told never to exceed six months. So, it was difficult for me and my baby, who would cry for hours and … I even wished I had not started at all … I got this information from the clinic. (Phakisi & Mathibe‐Neke, 2019, p. 31)

Moreover, HIV‐positive women were dissatisfied with the quality of infant feeding education and antiretroviral sessions. For instance, a lactating HIV‐positive woman from Malawi found sessions to be rushed with insufficient time spent on patient education: “It is because he didn't have enough time to explain what we are supposed to be doing; he seemed to be rushing too when he was conducting his sessions, yet we needed enough time to learn what we are supposed to be doing” (Katirayi et al., 2016, p. 5).

Women wanted information on formula or replacement feeding, not just breastfeeding advice alone (Horwood et al., 2018). Community education to inform antiretroviral use for breastfeeding so partners and families can facilitate their infant feeding experience was suggested by one HIV‐positive woman in order to reduce societal stigma (Chadambuka et al., 2018).

## 4 DISCUSSION

This is the first review to specifically synthesise HIV‐positive women's experiences of breastfeeding with antiretrovirals under the infant feeding and antiretroviral guidelines from 2010. The five analytical themes developed suggest HIV‐positive women have variable perspectives of breastfeeding with antiretrovirals, due to various levels of knowledge of breastfeeding and antiretrovirals, the emotionally challenging experience of breastfeeding with antiretrovirals and decision making, levels of disclosure of their HIV status, social stigmatisation, coping mechanisms and the level of support from partners, family members and health care professionals. We identified areas of support surrounding needs for role models and further education.

We identified some HIV‐positive women having awareness of protocols for breastfeeding with antiretroviral regimens, the potential risks of HIV MTCT and breastfeeding benefits. However, there were women who were unaware that they could breastfeed via antiretrovirals while being HIV positive. Similarly, Ladzani et al.'s (2011) and Ndubuka et al.'s (2013) cross‐sectional studies identified gaps in HIV‐positive women's infant feeding knowledge and awareness of existing guideline. Most infant feeding advice for HIV‐positive women concurred with infant feeding guidelines at the time when study data were collected; however, some women had been misinformed by health care professionals. Misinformation from health care professionals could reflect rapidly changing guidance on infant feeding advice (Nkwo, 2012). This is reflected by Chinkonde et al. (2010), whose in‐depth interviews with health care professionals on PMTCT care, found they struggled to advise breastfeeding with antiretrovirals and following national and local guideline changes to infant feeding in HIV‐positive women. The World Alliance for Breastfeeding Action (WABA) (2012) highlighted the need for a shift from infant feeding decision making to providing support for HIV‐positive women in their chosen infant feeding practice. Health care professionals should consistently update their knowledge and disseminate the most current national and local guidelines. Additionally, health care professionals require effective communication with HIV‐positive women to address their confusion on the risk of MTCT with breastfeeding. However, breastfeeding counselling interventions for HIV‐positive women have been found to have little success to encourage HIV‐positive women to exclusively breastfeed (Bosire et al., 2016). One suggestion for the lack of success might be due to the stigmatisation HIV‐positive women experienced from EBF (Bosire et al., 2016), but further exploration is needed.

Our review highlighted a lack of social support for HIV‐positive women to breastfeed due to nondisclosure of their HIV‐status to partners, family and community, often because of the socio‐cultural stigma surrounding nonbreastfeeding behaviours and taking antiretrovirals associated with HIV. This discouraged HIV‐positive women to breastfeed and adhere to protocols for breastfeeding with antiretrovirals. This finding has been reported in quantitative research. Maru et al. (2009) found a significant correlation between HIV disclosure and HIV‐positive women receiving partner support in infant feeding practices. In contrast, other quantitative studies have found no association between social support, stigma, disclosure of HIV/ART status and nonadherence and suggested associated individual factors, such as self‐efficacy and poor mental health could affect adherence and reduce social support (George & McGrath, 2018).

Our review found that HIV‐positive women seek role models among other HIV‐positive women breastfeeding, a potential area for future research as a way to enhance PMTCT care and promote breastfeeding with antiretrovirals in HIV‐positive women. Furthermore, our review indicated normalisation from breastfeeding with antiretrovirals overcoming social stigmatisation from appearing like noninfected women, particularly with Option B+. This warrants the need for further exploration into social stigma and understanding community views on antiretroviral use for breastfeeding to tackle stigma. Normality for HIV‐positive women and community education on HIV‐positive women breastfeeding with antiretrovirals are essential in avoiding potential socio‐cultural stigma. Educational interventions and community organisations have demonstrated to optimistically convert public views and reduce HIV related stigma (Pulerwitz et al., 2010).

HIV‐positive women experienced many emotions, particularly with infant feeding decision making, as their experiences were complex and reflected consideration of several factors, such as the risk of HIV MTCT, lifestyle and financial constraints, side effects of antiretrovirals, pressure from health care professionals and family to breastfeed and achieving motherhood. These could be key predictors into factors of breastfeeding initiation or duration and adhering to infant feeding protocol guideline. Nyoni et al.'s (2019) realist review on infant feeding counselling for HIV‐positive women concluded that EBF was linked to HIV‐positive women seeking positive feelings in motherhood. This highlights how counselling and psychosocial support services are essential in PMTCT care (WHO & UNICEF, 2016). Normalisation was a key factor in deciding to breastfeed with antiretrovirals, from satisfying emotional needs of achieving motherhood and infant bonding. This is reflected in Yezingane Network and UNICEF's (2011) infant feeding advice for HIV‐positive women in South Africa to promote breastfeeding. However, unpleasant side effects of antiretrovirals and the emotional challenges for HIV‐positive women to administer infant antiretrovirals were a deterrent for women to adhere to optimal breastfeeding protocol with antiretroviral regimens. This correlates with previous quantitative studies finding reduced antiretroviral adherence while breastfeeding compared to pregnancy (Haas et al., 2016; Nachega et al., 2012). This aligns with the need for interventions to adequately prepare women for Option B+, a lifelong commitment, as addressed by WHO and UNICEF (2016).

Managing to cope with breastfeeding with antiretrovirals involved individual characteristics of self‐efficacy and resilience from HIV‐positive women. Previous research has analogously shown HIV‐positive women with certain characteristics, such as resilience and motivation, to have greater antiretroviral adherence (Holstad et al., 2016) and when making infant feeding decisions (Morrison et al., 2011). However, Vrazo et al.'s (2017) systematic review on PMTCT interventions uptake amongst HIV‐positive pregnant and breastfeeding women questioned whether interventions for older infant feeding protocols were suitable for improving adherence and retention outcomes for pregnant and breastfeeding HIV‐positive women on Option B+, as only one study investigated interventions designed for Option B+. Further research is required on interventions for HIV‐positive women to improve retention and adherence to the safest antiretroviral regimen while breastfeeding. Religion was important for some women, correlating with Szaflarski's (2013) review on the importance of spirituality and religion on producing positive outcomes in HIV‐positive individuals.

### 4.1 Strengths and limitations

We conducted a robust search to identify all relevant existing literature, including all studies from any country and health care setting. All included studies were critically appraised by two researchers to improve rigour. A limiting factor is the ambiguity in deciphering whether studies explored breastfeeding in the context of antiretrovirals, as studies that did not mention antiretroviral use were not included in our review. All included studies were conducted in Africa, where HIV prevalence is high and limited resources for replacement feeding, limiting the transferability of the findings to populations of HIV‐positive women situated in other countries and settings. Future qualitative research on this topic in high‐income settings is needed to further the understanding of experiences of HIV‐positive women in settings where replacement feeding advice is promoted.

## 5 CONCLUSION

HIV‐positive women were challenged by fears over the risk of HIV MTCT, lifestyle factors, emotional needs and socio‐cultural factors when making infant feeding decisions and breastfeeding with antiretrovirals. HIV‐positive women were in receipt of variable levels of social support (family, community and health care professionals), utilising various coping methods depending on the individual's circumstances. With antiretrovirals, particularly Option B+, HIV‐positive women experienced normalisation by dissociating themselves from socio‐cultural stigma from being able to breastfeed. Socio‐cultural stigma largely persists to hinder optimal breastfeeding practices for HIV‐positive women. Support from family, communities and health care professionals helped women to breastfeed with antiretrovirals. Further research into interventions aiming to promote HIV‐positive women's adherence and commitment to lifelong ART for breastfeeding is required.

## CONTRIBUTIONS

KMCL conceptualised the review. KMCL and Y‐SC developed the review question and design with DB's input. The literature search was performed by KMCL under supervision from Y‐SC. KMCL, Y‐SC and KYCL conducted data extraction. Data analysis was performed by KMCL, Y‐SC and KYCL. Quality assessment was undertaken by Y‐SC and KMCL. The original draft was prepared by KMCL. The draft was critically commented by Y‐SC, KYCL and DB. All authors read and approved the final version of the manuscript.

## CONFLICTS OF INTEREST

The authors declare that they have no conflicts of interest.

## Data Availability

As this is a qualitative evidence synthesis, the original data are available from published articles included in the review. The references of the included studies are in the reference list.

## APPENDIX A SUMMARY OF ANTIRETROVIRAL AND INFANT FEEDING GUIDELINES FOR HIV‐POSITIVE WOMEN OVER THE YEARS

**Table:**

Guideline	Woman's antiretroviral recommendation	Infant antiretroviral recommendation	Infant feeding recommendation
(WHO Global Programme on AIDS & UNICEF, 1992) Consensus statement from the WHO/UNICEF Consultation on HIV Transmission and Breast‐Feeding, Geneva, 30 April ‐ 1 May 1992	Not mentioned	Not mentioned	• Breastfeed regardless of status. • In settings with greater risk of infant death due to infectious disease and malnutrition, breastfeeding is advised.
UNICEF et al. (1998) A review of HIV transmission through breastfeeding. UNICEF‐UNAIDS‐WHO. HIV and infant feeding.	Nil recommendation, but discussed trials of antiretroviral regimens and their effectiveness, due to trials ongoing.	Antiretroviral prophylaxis postnatally	• Replacement feeding if possible. • If breastfeeding, early cessation as early as possible, or expressed breast milk or wet nursing from a tested HIV negative woman.
WHO (2001) New Data on the Prevention of Mother‐to‐Child Transmission of HIV and their Policy Implications.	To have antiretroviral prophylaxis regimen (ZDV, ZDV + 3TC or nevirapine) as per local policy	To have antiretroviral regimen as per local policy	• Replacement feeding if AFASS (Acceptable, Feasible, Affordable, Sustainable, Safe) criteria met • Exclusive breastfeeding for 6 months with rapid weaning and replacement feeding
WHO et al. (2007) HIV and Infant Feeding. Update based on the Technical Consultation held on behalf of the Inter‐agency Task Team (IATT) on Prevention of HIV Infection in Pregnant Women, Mothers and their Infants. Geneva, 25–27 October 2006	Antiretroviral therapy for women's health or 7 day prophylaxis postpartum	Prophylaxis for 7 days post‐birth	• Exclusive breastfeeding for first 6 months if AFASS criteria not met • Breastfeeding cessation at 6 months if infant's diet is adequate • Complementary feeding from 6 months if AFASS criteria not met • Weaning over 2–3 days or up to 2–3 weeks maximum • Rapid weaning is not recommended
WHO (2010) Guidelines on HIV and infant feeding. 2010. Principles and recommendations for infant feeding in the context of HIV and a summary of evidence.	Lifelong antiretroviral therapy or antiretroviral prophylaxis throughout pregnancy until 1 week post finishing breastfeeding	Daily antiretroviral prophylaxis if woman is not on treatment, lasting until 1–2 weeks post finishing breastfeeding or 4–6 weeks if women is on antiretroviral treatment	• Exclusive breastfeeding until 12 months • Complementary feeding from 6 months • Wean over one month.
WHO (2013) Consolidated guidelines on the use of antiretroviral drugs for treating and preventing HIV infection. Recommendations for a public health approach. June 2013.	Option B: Lifelong ART for pregnant and breastfeeding women eligible for treatment (CD4 count ≤350 cells/mm^3^) Option B+: Lifelong ART regardless of CD4 cell count.	If infant is breastfeeding: antiretroviral prophylaxis (nevirapine) once a day from birth for 6 weeks If infant is replacement feeding: Prophylaxis NVP once daily or AZT twice daily for 4–6 weeks	• Exclusive breastfeeding until 12 months if adequate diet available • Complementary feeding from 6 months • Wean over one month period
WHO and UNICEF (2016) Guideline updates on HIV and infant feeding. The duration of breastfeeding and support from health services to improve feeding practices among mothers living with HIV.	Option B+	Infant receives daily NVP or AZT for 4–6 weeks	• Exclusively breastfeed for 6 months • Complementary feeding from 6 months while continuing breastfeeding for up to 24 months and beyond • One month weaning period
